# The impact of national values on the prevention and control of COVID-19: An empirical study

**DOI:** 10.3389/fpsyg.2022.901471

**Published:** 2022-09-09

**Authors:** Yanwei Lyu, Jinning Zhang, Yue Wang

**Affiliations:** ^1^School of Business, Shandong University, Weihai, China; ^2^School of Software and Microelectronics, Peking University, Beijing, China

**Keywords:** COVID-19, national values, heterogeneity analysis, empirical study, endogenous

## Abstract

The outbreak of COVID-19 at the end of 2019 has become the most devastating public health event of the 21st century. The different performances of governments and people in different countries and regions show that national values may play an important role in the prevention and control of COVID-19. Based on data from the seventh wave of World Values Survey (WVS-7) and the Human Freedom Index (HFI) report in 2020, three national value factors are extracted in this manuscript, including religious belief, government satisfaction and individual freedom. Then ordinary least squares regression (OLS) regression model is constructed to explore the influence of these three value factors on the prevention and control of COVID-19 and some heterogeneity analysis is implemented. The results show that religious belief and individual freedom significantly increased the COVID-19 infection rate, while government satisfaction significantly reduced the COVID-19 infection rate. The study findings have the ability to hold up after a range of robustness. For countries and regions with different COVID-19 testing policies, the influence of national values is different. Only in countries and regions with high testing rate policies and complete systems of the prevention and control of COVID-19, the influence of national values is significant. Based on these findings, a series of targeted policy recommendations for building national values in the post-epidemic era are proposed.

## Introduction

At the end of 2019, COVID-19 swept the world. It has had a huge impact on the world’s politics, economy and people’s lives (Açikgöz and Günay, 2020; Liu K. et al., 2020). According to real-time statistics from the World Health Organization (WHO), as of August 6, 2021, the cumulative number of confirmed cases worldwide has exceeded 200 million. This represents 2.60% of the world’s total population and is very widespread. A variety of responses to COVID-19 have been adopted by various countries and regions, but the results are different. The reasons for this may be related to the objective level of development of each country. Existing studies have shown that poverty exacerbates the spread of infectious diseases (Goscé and Johansson, 2018), that population density is directly proportional to the rate of epidemic transmission (Qi et al., 2020), and that advanced medical equipment and services contribute to the effectiveness of epidemic prevention and control (Wang et al., 2021). However, it is noteworthy that China, as a developing country, outperforms some developed countries in Europe and the United States in terms of epidemic prevention and control effectiveness. In the case of China and the United States, for example, as of 6 August 2021, the United States had the highest number of infections in the world. It accounted for approximately 18.43% of the total COVID-19 confirmed cases. In contrast, the cumulative confirmed cases in China accounted for only 0.05%, though it is the country with the earlier start of the epidemic and the largest population base. In addition, the number of diagnosed cases per million people in the United States was already 393.76 times higher than in China^1^. This result is obviously difficult to be well explained by the objective development level of economic and medical facilities. In this context, this manuscript cannot help but consider the huge differences that subjective factors may have on the effectiveness of COVID-19 prevention and control across countries. In the case of China and the United States, for example, there are significant cultural differences between the two countries. As pointed out by Zhao (2008), China is primarily an Eastern Confucian culture, while the United States reveres Western culture, especially Greco-Roman culture. Within this cultural context, China emphasizes collectivism and focuses on group interests, while the West emphasizes individualism and focuses on individual interests. That is, the national values of the two countries have become more different. This manuscript therefore has reason to suspect that it is the different national values of each country and region that may lead to significant differences in governmental practices in response to the new epidemic, as well as different levels of national acceptance and implementation of the same epidemic prevention and control policies. Further, it has led to different epidemic prevention and control outcomes.

Individual values refer to a person’s belief and attitude system, while national values are based on the value expectations, behavioral guidance, and evaluation criteria agreed upon by the citizens of a country, and include religious, political, family, work, and cultural identity values, including religious, political, family, work, cultural identity and other aspects of national values. In the process of forming national values, they are influenced by factors such as historical traditions, cultural backgrounds and the realities of the country, so that national values vary from country to country.

Due to the wide coverage of national values, metrics are often used to quantify specific indicators in a certain aspect. Some scholars design corresponding metrics to quantify indicators according to their own research needs. For example, Van Hooft and De Jong (2009) developed a 7-item metrics to calculate the individualistic tendency of the respondents. Dorfman and Howell (1988) used a 6-item metrics to calculate collectivist tendencies, and Carlson et al. (2009) used a 6-item metrics to explore work-family balance. Some scholars use the World Values Survey data to quantify indicators. For example, Shi (2018) used the World Values Survey to assign scores to democratic cognition and democratic satisfaction, so as to measure the degree of democracy. Using the question “trust in people in the society” in the table, Hou et al. (2021) quantified the level of social trust. Huang and Guo (2019) characterize the gender inequality perspective with the help of seven questions from the World Values Survey (WVS) questionnaire on whether men are superior to women in terms of rights, opportunities, values, and dignity.

In addition, national values, as a microcosm of the subjective cognition of the people of a country, will be externalized into specific behaviors, resulting in specific social phenomena or institutional manifestations, which are related to the development of the country in many aspects. For a country’s economic development, values determine the mode of production to a certain extent, and only by maintaining the values of scientific and sustainable development can we promote the long-term development of the national economy for the better (Han, 2017). For business, national values influence business goals and investment intentions (Harris and Carr, 2008), as well as the importance companies place on brand culture (Sumaco et al., 2014). In terms of political stability, studies have shown that individualism is negatively related to a country’s political stability (Ezcurra, 2021). In foreign exchange, only by understanding and respecting each other’s values can cross-cultural communication proceed smoothly and international business activities will not be hindered by culture clashes (Jiang, 2020). National values also have a significant impact on people’s own health, for example, smokers are more likely to quit in countries that promote autonomy, equality, and harmony (Louise and Hassan, 2015). In addition, the level of democracy in a country affects atmospheric pollution (Shen and Chen, 2021), ecological values affect the public’s environmentally responsible behavior, and national values also affect the acceptance of the e-commerce and sharing economy (Manjul et al., 2019). In summary, national values can have a significant impact on the economy, politics, diplomacy and personal behavior, so we fully believe that national values will also play a role in the prevention and control of the COVID-19.

From the existing studies, scholars have mainly explored the impact of values on three aspects of the nation’s psychological profile, behavioral characteristics and the speed of transmission of COVID-19. On the psychological side, Federico et al. (2021) concluded that group satisfaction increases national solidarity against COVID-19, while national collective narcissism (i.e., nationals believe that groups within the state are entitled to privileges that are not recognized by nationals of other countries) has a negative impact. In addition, Long et al. (2021) argue that individual power values that emphasize individualism may increase anxiety associated with COVID-19, whereas collectivism and a higher sense of national identity may reduce anxiety about COVID-19. In terms of behavioral characteristics, Kenneth et al. (2020) states that measures such as lockdowns and quarantines to control COVID-19 can only work if the public trusts the government. A group of scholars represented by Atalay and Solmazer (2021) explored the relationship between Schwartz’s cultural values and national mobility during the COVID-19 period. They showed that hierarchy is the main factor in reducing national mobility, after controlling for the severity of the economy and disease. As for the spread of the epidemic, the first peak in the number of infections is reached more quickly in countries and regions with higher levels of social trust (Min, 2020). And individualism can also accelerate the spread of COVID-19. Neupane (2020) argued that it is individualism that makes COVID-19 more severe. In addition, egalitarianism and the degree of democracy seem to have played a counterproductive role in the prevention and control of this epidemic (Vadlamannati et al., 2021). However, the relationship between the level of freedom of the political system and the rate of COVID-19 infection is not significant (Mayer et al., 2020).

Existing research has confirmed the influence of national values on the prevention and control of the COVID-19 to a certain extent, but the following problems still exist. Firstly, existing studies on the factors influencing the prevention and control of COVID-19 have mainly focused on objective conditions, such as nucleic acid testing policies, medical care and income levels in various countries and regions, and less literature has addressed the effect of subjective perceptions of national values on the prevention and control of COVID-19. Secondly, the existing literature is limited in terms of national values indicators. National values are a highly comprehensive concept, and existing studies mainly focus on a few indicators such as collectivism, individualism, democratic importance and group satisfaction, while the research on other aspects is relatively lacking. Thirdly, existing studies on the impact of national values on the prevention and control of COVID-19 have mainly focused on qualitative studies based on case studies, and quantitative analyses using econometric regression models are extremely rare, which makes the accuracy and reliability of the research findings greatly reduced. Based on data from the seventh wave of WVS-7 (WVS-7, 2020) and the Human Freedom Index (HFI) report in 2020 (Vásquez and McMahon, 2020), this manuscript extracts three national value factors. They are religious belief, government satisfaction and individual freedom. This manuscript then constructs an econometric regression model to explore the impact of these three values variables on the prevention and control of COVID-19. Furthermore, this manuscript conducts a heterogeneity analysis and finally proposes targeted policy recommendations. It is hoped that this manuscript can provide policy insights and decision-making basis for the prevention and control of COVID-19 in various countries and regions around the world.

**FIGURE 1:**
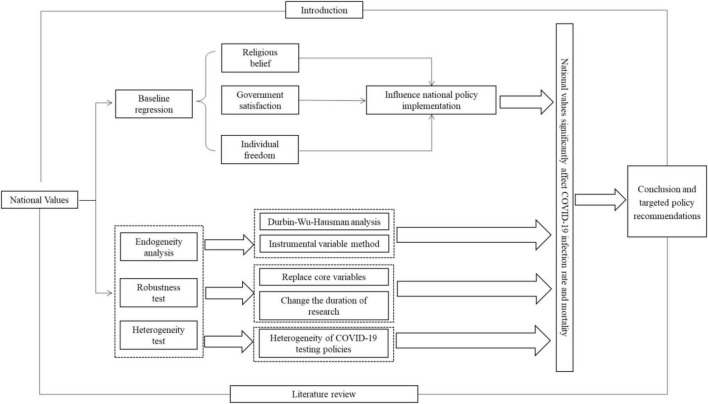
Research framework.

The rest of the manuscript is organized as follows. Mechanism analysis is shown in section “Mechanism analysis”. Methodology and data used in this study is included in section “Methodology and data”. In section “Empirical analysis”, the empirical results are presented and discussed. In section “Conclusion and policy recommendations”, the conclusions and corresponding policy implications are provided. The specific research framework is shown in Figure 1.

## Mechanism analysis

### Religious belief

Religious belief is likely to be detrimental to the prevention and control of COVID-19. That is, high religiosity of a country’s citizens and more religious adherents are negatively related to the prevention and control effect of COVID-19. On the one hand, frequent religious gatherings will occur in countries with a high number of religious believers. This will increase the probability of aggregated infections and thus affect the effectiveness of COVID-19 control. In South Korea, for example, there have been numerous mass infections of churches in the “metropolitan area”, centered on Seoul, since March 2020 (Ding, 2021). On the other hand, national acceptance and implementation of government policies may be influenced by religious belief (Marušić and White, 2018). As religious believers need to attend church regularly, they may object to policies such as home segregation and avoidance of gatherings. Nationals in places such as the United States even marched in protest, which could affect the effectiveness of the policies.

### Government satisfaction

Increased government satisfaction might be beneficial to the control effectiveness of COVID-19. This may be due to the fact that increased government satisfaction is accompanied by increased trust in the government and its decisions. Accordingly, government policies such as home quarantine and reduced mobility will be more consciously implemented by the population (Bargain and Aminjonov, 2020). These are undoubtedly beneficial to the effectiveness of COVID-19 control. Of course, if a country’s government makes poor decisions, higher government satisfaction may be detrimental. However, the reason why this conclusion was not reached in this manuscript may be due to the fact that government satisfaction also reflects some objective capabilities of the government. The United Kingdom government’s trust has declined as a result of a surge in the number of infections due to the introduction of herd immunization (Newton, 2020). Therefore, the high level of government satisfaction is also a reflection of the government’s responsiveness, ability to deliver, and the availability of healthcare resources. This may also help the country to achieve better results in COVID-19 control.

### Individual freedom

Individual freedom may be negatively related to the prevention and control effect of COVID-19. This means that the more people pursue individual freedom, the higher the infection rate of COVID-19. On the one hand, individual freedom advocates believe that individuals have an unsurpassed right to freedom in relation to the state and government. For this reason, they refuse to wear masks and often hold gatherings to protest, making government control initiatives ineffective and further increasing numbers of infections (Qi and Gao, 2021). On the other hand, citizens who advocate individualism pay more attention to personal interests and will not sacrifice their economic interests to fight the epidemic, which is not conducive to epidemic prevention and control (Lin, 2021). The fight against COVID-19 requires the solidarity and self-giving of the population. But for the individual freedom advocates, it is difficult to sacrifice individual interests for the collective good. Therefore, the impact of individual freedom should be theoretically negative.

## Methodology and data

### Regression model

This manuscript uses an econometric regression model to explore the impact of national values on the prevention and control of COVID-19, and the model is expressed as follows:


(1) $$c\,o\,v\,i=\beta_{0}+\beta_{1}\,r\,e\,l\,i+\beta_{2}\,g\,o\,v\,e+\beta_{3}\,f\,r\,e\,e+\beta_{4}\,X_{i}+\varepsilon$$

Where *covi* indicates COVID-19 infection rate, *reli, gove*, and *free* are the three national values variables in this manuscript, representing religious belief, government satisfaction and individual freedom, respectively, *X*_*i*_ indicates a set of control variables including healthcare access and quality index (*X*_1_), smoking prevalence among the population aged 15 and over (*X*_2_), government stringency index (*X*_3_) and level of economic development (*X*_4_), and ε is random error term.

### Variables and data

Explained variables. COVID-19 infection rate (*covi*). It is the logarithm of the cumulative confirmed COVID-19 cases for each country and region from the first reported case to 6 August 2021^2^. The main data sources currently monitoring and publishing confirmed cases of COVID-19 worldwide are the WHO, European Centre for Disease Prevention and Control (ECDC) and The Johns Hopkins University. The COVID-19 infection rate variable in this manuscript was derived from Our World in Data, created by the University of Oxford. This is because there are differences in the number of confirmed COVID-19 cases between the different data sources, but Our World in Data combines these three sources with some corrections, so the data is more detailed and more authoritative.

Explanatory variables. National values, including religious belief (*reli*), government satisfaction (*gove*), and individual freedom (*free*). In the selection of explanatory variables, in order to cover as many variables of national values as possible, the WVS database is taken as the starting point of this manuscript. This manuscript tries to screen out all exogenous value variables that may be related to the prevention and control of COVID-19 from the database. Then factor analysis method is used to extract and summarize the two factors: religious belief and government satisfaction. These are the two explanatory variables of this manuscript. At the same time, because many variables representing liberalism and democracy in the database are missing in many countries and regions, this manuscript uses the individual freedom in the Human Freedom Index (HFI). In this way, the full coverage of national values variables can be achieved. It is worth noting that the three explanatory variables are mainly related to a country’s cultural concepts and historical evolution. They are hardly affected by the level of objective economic development. So they are exogenous and reliable. Specifically, religious belief mainly measures the degree of a country’s citizens’ belief in and implementation of religious creeds and concepts. It represents national belief and spiritual choice. Government satisfaction refers to the national support and self-confidence for the government. It shows the political position and collective identity of the people. Individual liberalism means that citizens pursue their own freedom and personal interests. This concept is opposite to collectivism.

**FIGURE 2:**
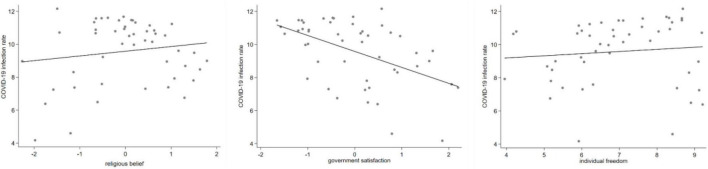
Scatter plot of national values on the COVID-19 infection rate.

Specifically, the variables of religious belief and government satisfaction are derived from the (2017-2020) (WVS-7). The dataset, surveyed and published by The World Values Survey Association (WVSA), includes 14 themes and over 300 indicators, with data available for 49 countries and territories worldwide, covering 129,000 respondents. In this manuscript, seven value questions related to religious belief and government satisfaction are selected from this dataset. And each question is assigned a score on a percentage scale. Then the principal component factors are extracted as explanatory variables through principal components analysis (PCA) and the kaiser varimax (Kaiser, 1958). In addition, the value of the KMO statistic calculated in this manuscript is 0.750 and Bartlett test is significant, indicating that the correlation between the original variables is strong and the original variables are suitable for extracting the principal component factors^3^. The results of PCA are shown in Table 1. Figure 2 shows the scatter plot of national values on the COVID-19 infection rate.

**TABLE 1:** Results of principal components analysis (PCA).

		First principal component factor	Second principal component factor
Characteristic values		3.662	2.475
Variance contribution rate		52.316%	35.363%
Principal component load matrix	Importance of religion	0.959	–0.187
	Satisfaction with the political system performance	–0.086	0.947
	Respect for individual human rights by the government	–0.260	0.895
	Trust in religion over science	0.915	–0.026
	Believe in God	0.834	–0.385
	Confidence of the government	0.123	0.943
	Confidence of the Churches	0.883	0.211
Naming of principal component factors		Religious	Government
		belief	satisfaction

As can be seen from the table, the first principal component factor has large loading coefficients on four indicators. They are importance of religion, trust in religion over science, believing in God and confidence of the Churches, which are named as the religious belief. The second principal component factor has a large loading coefficient on three indicators, including satisfaction with the political system performance, respect for individual human rights by the government and the confidence of the government, naming it the government satisfaction.

The individual freedom variable is derived from the HFI in 2020, published jointly by the Cato Institute in the United States and the Fraser Institute in Canadian. It uses 79 distinct indicators of personal and economic freedom in 12 areas. The areas include Rule of Law, Security and Safety, Movement, Religion, Association, Assembly, and Civil Society, Expression and Information, Identity and Relationships, Size of Government, Legal System and Property Rights, Access to Sound Money, Freedom to Trade Internationally, Regulation of Credit, Labor and Business. What’s more, it is the most comprehensive freedom index created for a globally meaningful set of countries. The individual freedom variable used in this manuscript is the HFI in this report.

Control variables. In terms of the selection of control variables, existing studies show that the prevention and control effect of COVID-19 will be mainly affected by the national medical level (Wang et al., 2021), demographic characteristics (Xu et al., 2020), economic development level (Liu L. et al., 2020), government prevention and control measures (Maier and Brockmann, 2020), and other objective factors. Combined with the availability of data and the exogenous of variables, this manuscript finally selects an index in each of the above four aspects as the control variables. So we introduce the following control variables: (1) healthcare access and quality index (*X*_1_); (2) smoking rate of population over 15 years old (*X*_2_); (3) government stringency index (*X*_3_) and; (4) level of economic development (*X*_4_).

The healthcare access and quality index is derived from The Global Burden of Disease Study (GBD), published jointly by the WHO and Institute for Health Metrics and Evaluation, University of Washington, in 2020. The indicator is based on mortality from 32 causes of death (also known as affordable mortality) that could have been prevented by timely and effective medical care. The smoking prevalence among the population aged 15 and over is derived from the University of Oxford’s Our World in Data. This database calculates the number of people aged 15 and over who smoke as a proportion of the total population in each country and region in 2020. The government stringency index is derived from Oxford COVID-19 Government Response Tracker (OxCGRT), launched by the University of Oxford in 2020. This system was initiated by the Blavatnik School of Government at Oxford University. It is the world’s first tool to track and compare in real time the response policies of governments around the world to the development of COVID-19. To date, the system has tracked information on the various responses to COVID-19 and the development of the epidemic in over 190 countries and regions to reflect the level of action taken by each country and region in response to COVID-19. The level of economic development variable is characterized by the GDP per capita of each country and region in 2020, which is sourced from the World Bank (Wang et al., 2019).

### Descriptive statistics

This manuscript uses the 49 countries and regions covered by the WVS-7 as its sample. The data are obtained from Oxford University’s Our World in Data, the WVS-7, the GBD report, the HFI report in 2020, and OxCGRT, etc. The descriptive statistics for each variable are shown in Table 2.

**TABLE 2:** Descriptive statistics of variables.

Variable	Symbol	N	Mean	SD	Min	Max
COVID-19 infection rate	*covi*	49	9.5791	1.9352	4.1756	12.1653
Religious belief	*reli*	49	0	1	–2.2629	1.7843
Government satisfaction	*gove*	49	0	1	–1.6551	2.1950
Individual freedom	*free*	49	6.9585	1.4555	3.95	9.21
Healthcare access and quality index	*X*_1_	49	68.8469	12.8550	43.1	94.6
Smoking prevalence among the population aged 15 and over	*X*_2_	49	0.2313	0.1008	0.044	0.434
Government stringency index	*X*_3_	49	56.1988	17.4724	2.780	90.740
Level of economic development	*X*_4_	49	2.0613	1.8619	0.1730	10.4862

## Empirical analysis

### Baseline regression analysis

Before performing the baseline regression, the model is first tested for multicollinearity using the variance inflation factor (VIF), the results are shown in the second column of Table 3. It can be seen that the VIF of each variable in the model does not exceed 5, indicating that the problem of multicollinearity is negligible and the econometric regression model is reasonably set (Kim, 2019). Ordinary least squares regression (OLS) is used to test the effect of the three national values variables on the COVID-19 infection rate, and the results are shown in Table 3. The table shows that the coefficient of influence of religious belief on the COVID-19 infection rate is 1.2995 and is significant at the 1% level. This indicates that religious belief may increase the COVID-19 infection rate. The coefficient of influence of government satisfaction factor on COVID-19 infection rate is −0.8347 and significant at the 1% level. This indicates that government satisfaction might reduce the prevalence of COVID-19 infection. The coefficient of influence of individual freedom is 0.4262 and is significant at the 5% level. This indicates that the prevalence of COVID-19 infection is positively correlated with individual freedom and individual freedom may promote the deterioration of the spread of COVID-19, resulting in socio-economic and livelihood damage to the entire country and region (Kernohan, 1998).

**TABLE 3:** Baseline regression estimation results.

Variable	VIF	Coefficient	*t*
*reli*	3.5296	1.2995***	3.4878
*gove*	1.1432	−0.8347***	–3.9365
*free*	2.4368	0.4262**	2.0038
*X*_1_	3.6256	0.0830***	2.8266
*X*_2_	1.1531	4.4118**	2.0874
*X*_3_	1.1384	0.0285**	2.3598
*X*_4_	2.0092	–0.2417	–1.6008
*N*	49		
*F*-statistic	9.32		
*R*-squared	0.5694		
Adj *R*-squared	0.4959		

***, **, and * significance at 1, 5, and 10% levels, respectively.

From the empirical results of this manuscript, COVID-19 infection rate is positively associated with religious belief at the 1% significance level. That is, the higher the religious belief of a country’s population, the more religious believers there are, and the worse COVID-19 control effective is going to be. Of course, this impact is not absolute. Religious groups in very few countries are willing to help and cooperate with the government’s policies in this regard. The Church of England, for example, shifted relevant religious activities online and closed all churches (Pan, 2021), without negatively affecting the overall epidemic control. The Holy See, for its part, set up an epidemic commission to provide the world with an economic and social recovery plan for the post-epidemic era, with “holistic ecology” at its core (Xiao, 2021). This has, to some extent, contributed to the COVID-19 control in Rome. Globally, however, this type of religion is still a minority. Therefore, the empirical results of this manuscript are, on the whole, more than reasonable. The results of the baseline regression suggest that increased government satisfaction is beneficial to the control effectiveness of COVID-19, but Newton (2020) also points out that in major events that pose a direct impact on the lives of individuals, the practices of the public may be less susceptible to the influence of government, or other organizations. However, people may not be able to find the right personal response to new outbreaks of disease such as COVID-19 in a short period of time, and that government policies and directives may have an impact on their personal perceptions. Therefore, the manuscript’s conclusion that government satisfaction is negatively associated with COVID-19 prevalence is still relatively convincing. Empirical results have shown that individual freedom significantly increases the COVID-19 infection rate, the impact of individual freedom should be theoretically negative, and our results confirm this conclusion, which is similar to the results of other studies.

The regression coefficients of the control variables show that healthcare access and quality index may have a significant positive effect on the prevalence of COVID-19 infection. This may be due to the fact that countries with better healthcare access and quality index have a higher probability of nationals being diagnosed with COVID-19 infection and a higher rate of COVID-19 infection. There is positive correlation between the smoking prevalence among the population aged 15 and the prevalence of COVID-19 infection. This may be due to the fact that smoking causes severe damage to the body’s immunity and makes the body more susceptible to infectious types of diseases such as COVID-19 in the presence of a reduced immunity (Qiu et al., 2017). The government stringency index is likely to have a significant positive effect. This may be because the more rapidly COVID-19 spreads and the more severe the situation, the more the government is forced to adopt a higher response level (Zhou and Coleman, 2016). So, the COVID-19 infection rate will increase during this period. The effect of the level of economic development is not significant. This shows that the level of economic development may not be linearly related to the infection rate of COVID-19, and there may be a more complex impact mechanism.

### Endogenous processing

The above studies suggest that national values can influence COVID-19 infection rates. At the same time, high COVID-19 infection rates may in turn reshape national values. This suggests that there may be a bidirectional causal relationship between national values and COVID-19 prevalence. This may affect the accuracy of the OLS regression coefficients. Therefore, exploring the effect of national values on the COVID-19 infection rate requires addressing the possible reverse causality. This manuscript utilizes a heteroscedasticity robust Durbin-Wu-Hausman (DWH) test for endogeneity. The principle is to first regress the endogenous variables on the exogenous and instrumental variables and calculate their residuals. The residuals are then put into an OLS regression model as explanatory variables to examine whether the coefficient on the residuals is zero (Patrick, 2021). The results of this manuscript reject the original hypothesis that all explanatory variables are exogenous at the 10% significance level. Therefore, the national values variables are endogenous.

The most common way to address endogeneity is to select the appropriate instrumental variable. For the selection of instrumental variables, conflict and terrorist attack mortality in a country and region (*terr*) is selected as the instrumental variable for religious adherence. This is because regional conflicts and terrorist attacks tend to be strongly associated with extreme religious ideology (Saiya, 2019), but not with COVID-19 infection rates. The drowning mortality for a country and region (*drow*) is selected as an instrumental variable for government satisfaction. This is because the drowning mortality in a country and region is highly related to the safety and security facilities provided by the government (Brooks, 2005), which will affect the national satisfaction with the government. However, drowning mortality does not affect COVID-19 infection rates. The geographic location of countries and regions (*geog*) is chosen as an instrumental variable for individual freedom. This is because individual freedom is the most fundamental and orthodox system of social thought in Western countries and regions. The values of individual supremacy and freedom are deeply embedded in the minds of the nationals of Western countries. Asian countries and regions, on the other hand, advocate the value of collectivism. They emphasize the importance of collective interests over individual interests. Their citizens prefer to pursue individual freedom in the context of a collective code of conduct (Gouveia et al., 2002). However, the geographical location of countries and regions is naturally present and generally does not change and does not affect COVID-19 infection rates. Therefore, this manuscript introduces a dummy variable for geographic location. This manuscript sets the value of 1 for countries and regions in Europe, the Americas and Oceania, while setting the value of 0 for countries and regions in Asia. In summary, all of the above variables satisfy both the exogeneity assumption and are correlated with the explanatory variables, and it is appropriate to use these variables as instrumental variables.

In addition, we need to test for the validity of the instrumental variables. A rule for testing weak instrumental variables is that if the value of the *F*-statistic is greater than 10 in the first stage of the regression, there is no need to worry about weak instrumental variables (Bound et al., 1995). So, this manuscript re-estimates the three instrumental variables of conflict and terrorist attack mortality, drowning mortality and geographic location separately into the model. The results show that first stage *F*-statistics are 39.70, 5.88, and 20.77 for these three instrumental variables, respectively. So drowning mortality suffers from the problem of weak instrumental variables. Therefore, this manuscript draws on the study by Combes et al. (2019) to estimate the regression results for this instrumental variable using the limited information maximum likelihood method (LIML) (Model 2 in Table 4). In contrast to the method of two-stage least squares (2SLS), LIML is insensitive to weak instrumental variables. Therefore, even in the presence of weak instrumental variables, the LIML estimates are less affected by the presence of weak instrumental variables.

**TABLE 4:** Regression estimation results for instrumental variables.

Variable	Model 1 (2SLS)		Model 2 (LIML)		Model 3 (2SLS)	
	First-stage	Second-stage	First-stage	Second-stage	First-stage	Second-stage
*reli*		4.0957** (2.0861)				
*gove*				−1.5554*** (−3.4764)		
*free*						1.9559** (2.0776)
*terr*	0.0046* (1.8017)					
*drow*			0.2433*** (2.8026)			
*geog*					0.8988*** (2.7827)	
*X*_1_	−0.0534*** (−6.7239)	0.2788** (2.4333)	0.0029 (0.1525)	0.0195 (0.7694)	0.0403*** (2.7889)	−0.0564 (−0.8199)
*X*_2_	0.7068 (0.8778)	1.1507 (0.3299)	0.6398 (0.4093)	6.1773*** (2.7669)	0.0172 (0.0109)	3.6240 (0.9420)
*X*_3_	−0.0036 (−0.6685)	0.0269* (1.9115)	0.0049 (0.7038)	0.0281** (2.2883)	−0.0019 (−0.2497)	0.0283 (1.5256)
*X*_4_	−0.1279*** (−2.9888)	0.0761 (0.2719)	0.1138 (1.4844)	−0.1971 (−1.2587)	0.2694*** (3.4781)	−0.8546*** (−4.1749)
N	49	49	49	49	44	44
*F*-statistic	39.70		5.88		20.77	
*R*-squared	0.6838		0.2879	0.3471	0.6171	

***, **, and * indicate significance at 1, 5, and 10% levels, respectively, and those in () are the t-values of the corresponding test statistics.

Models 1, model 2, and model 3 in Table 4 report the results of re-estimating the three instrumental variables of conflict and terrorist attack mortality, drowning mortality, geographic location separately in the model. The table shows the sign direction of the estimated coefficients of religious belief, government satisfaction and individual freedom are fully consistent with the baseline regression results after using instrumental variables. Moreover, they are significant at the 5, 1, and 5% levels, respectively. This confirms the accuracy of the findings of the previous study. However, the estimated coefficients for the three values variables obtained from the instrumental variables approach were significantly larger than the OLS estimated coefficients. This suggests that the OLS estimates may underestimate the true effect of the three values variables on COVID-19 infection rates due to endogeneity, thus causing attenuation bias in the estimated coefficients. The instrumental variables approach addresses this issue to some extent, making the estimates more credible.

### Robustness tests

#### Substitution of explanatory variables

As the religious belief and government satisfaction used in this manuscript are public factors extracted through PCA and are not raw values data, there may be problems with data distortion. Therefore, the original values variable with the highest correlation with each of these two principal component factors are selected separately and re-run as a replacement variable. The two original values variables are the importance of religion (reli*), and satisfaction with the political system performance (gove*). In addition, we also re-run the regression with the geographic location dummy variable (free*) as a replacement variable for individual freedom. That is setting the value to 1 for countries and regions in Europe, the Americas and Oceania, and 0 for countries and regions in Asia. The regression results are shown in Table 5.

**TABLE 5:** Regression results with replacement of explanatory variables.

Variable	Model 1	Model 2	Model 3
*reli*		1.4795*** (4.3730)	
*gove*		−0.6962*** (−2.8583)	
*free*	0.5013** (2.1868)		
reli*	0.0514*** (3.2249)		0.0565*** (4.0631)
gove*	−0.0397** (−2.2385)		−0.0342* (−1.7796)
free*		1.2837** (2.4670)	1.4840*** (2.9079)
*X*_1_	0.0723*** (2.6741)	0.0899*** (3.3361)	0.0709*** (2.9227)
*X*_2_	4.1523* (1.8809)	3.8350* (1.8907)	3.8025* (1.8459)
*X*_3_	0.0283** (2.2063)	0.0321*** (2.7259)	0.0332*** (2.7347)
*X*_4_	−0.2955* (−1.8706)	−0.0876 (−0.6513)	−0.1009 (−0.7101)
*N*	49	44	44
*R*-squared	0.5210	0.6657	0.9437
Adj *R*-squared	0.4392	0.6007	0.5745

***, **, and * indicate significance at 1, 5, and 10% levels, respectively, and those in () are the t-values of the corresponding test statistics.

Whether only the substitution variables for religious belief and government satisfaction are included in the model (Model 1 in Table 5), or only the substitution variable for individual freedom is included in the model (Model 2 in Table 5), or the substitution variables for all three value variables are (Model 3 in Table 5), the regression results show that religious belief and individual freedom significantly increase COVID-19 prevalence, while government satisfaction significantly decreases COVID-19 prevalence. It can be seen that after replacing the explanatory variables, although the magnitude of the regression coefficients change compared to the baseline regression results, the direction of sign is exactly the same as the baseline regression results. The regression coefficients are all highly significant and the regression results are robust.

In addition, the regression coefficients for geographical location, a proxy variable for individual freedom, suggest that geographical location factors contribute to an average difference of 1.3839 percentage points in the logarithm of COVID-19 infection rates. Countries and regions in Europe, the Americas and Oceania that promote individualistic values are higher than those in Asia that promote collectivistic values^4^. This suggests that Western-style individual freedom significantly increases the rate of COVID-19 infection compared to Asian countries and regions that promote collectivist values. As a result, the spread of COVID-19 worsens and the common freedom rights of social groups are compromised. This ultimately affects the daily lives and health of the entire population (Morand and Walther, 2018).

#### Substitution of explained variables

In this manuscript, the explained variables are replaced with COVID-19 mortality, which can also be used to represent the effect of COVID-19 prevention and control. Then the regressions are run again, and the results are shown in Table 6.

**TABLE 6:** Regression results with replacement of explained variables.

Variable	Model 1	Model 2	Model 3	Model 4
*reli*	1.0081*** (2.7561)		1.1946*** (3.7666)	
*gove*	−1.0844*** (−5.2097)		−0.8061*** (−3.5304)	
*free*	0.5066** (2.4262)	0.5832** (2.5379)		
reli*		0.0405** (2.5390)		0.0453*** (3.4642)
gove*		−0.0591*** (−3.3272)		−0.0445** (−2.4614)
free*			1.7216*** (3.5295)	1.9441*** (4.0517)
*X*_1_	0.0535* (1.8559)	0.0521* (1.9225)	0.0592** (2.3429)	0.0457** (2.0034)
*X*_2_	3.7515* (1.8081)	3.2897 (1.4864)	2.3553 (1.2387)	2.1495 (1.1098)
*X*_3_	0.0377*** (3.1702)	0.0379*** (2.9494)	0.0422*** (3.8266)	0.0435*** (3.8137)
*X*_4_	−0.3065** (−2.0677)	−0.3820** (−2.4123)	−0.1155 (−0.9164)	−0.1405 (−1.0524)
*N*	49	49	44	44
*R*-squared	0.6172	0.5559	0.7274	0.7077
Adj *R*-squared	0.5518	0.4801	0.6744	0.6509

***, **, and * indicate significance at 1, 5, and 10% levels, respectively, and those in () are the t-values of the corresponding test statistics.

The regression results for either model 1, model 2, model 3, or model 4 again show that religious belief and individual freedom significantly increase COVID-19 mortality, while government satisfaction significantly decreases COVID-19 mortality. Although the magnitude of the regression coefficients of the three value variables (or replacement variables) changes after replacing the explanatory variables compared to the baseline regression results, the direction of sign is exactly the same as the baseline regression results and the regression coefficients are all highly significant. This indicates that the regression results are robust.

In addition, the regression coefficients for geographic location, a proxy variable for individual freedom, show that geographical location factors contribute to an average difference of 1.8329^5^ percentage points in the logarithm of COVID-19 mortality. Countries and regions in Europe, the Americas and Oceania that promote individualistic values are higher than those in Asia that promote collectivistic values. This suggests that Western-style individual freedom significantly increases not only COVID-19 infection rates but also COVID-19 mortality rates compared to Asian countries and regions that promote collectivist values (Lee, 2020). Moreover, the increase in COVID-19 mortality is greater than the increase in COVID-19 infection. Therefore, COVID-19 prevention and control requires strict prevention of excessive individual freedom behavior. The government should promote the development and practice of proper collectivist values among the population. Only if people follow a collective code of conduct can they be freed from the threat of exposure to COVID-19 more quickly.

#### Changing the time point of the study

The time point for this study is from the first reported case in each country and region to 6 August 2021 (the first time that Cumulative confirmed COVID-19 cases worldwide exceeds 200 million). To avoid chance in the choice of time points, the manuscript further changes the time points of the study to two time periods. The first time period is from the first case reported in each country and region to 27 January 2021 (when the cumulative number of confirmed COVID-19 cases worldwide exceeded 100 million for the first time), and the second time period is from 28 January 2021 to 6 August 2021. In this manuscript, regression analyses are conducted separately using these two time periods as the study period to examine the robustness of the study findings. The regression results are shown in Table 7.

**TABLE 7:** Regression results for changing study time points.

Variable	The first case reported to 27 January 2021		28 January 2021 to 6 August 2021	
	Model 1	Model 2	Model 3	Model 4
*reli*	1.0333** (2.4190)		1.6677*** (3.8457)	
*gove*	−0.9623*** (−4.0641)		−0.9231*** (−3.7401)	
*free*	0.3558* (1.7006)		0.5415** (2.1874)	
reli*		0.0442*** (2.7368)		0.0684*** (4.0153)
gove*		−0.0381* (−1.7464)		−0.0420* (−1.7829)
free*		1.7444*** (3.1370)		1.3759** (2.2007)
*X*_1_	0.0537 (1.5883)	0.0338 (1.1930)	0.0926*** (2.7092)	0.0803*** (2.7030)
*X*_2_	3.4588 (1.4359)	2.3207 (0.9835)	4.8609** (1.9760)	4.2070* (1.6670)
*X*_3_			0.0374** (2.6547)	0.0403*** (2.7118)
X_3_*^a^	0.0300** (2.3282)	0.0343*** (2.7626)		
*X*_4_	−0.1071 (−0.6350)	0.0114 (0.0725)	−0.2771 (−1.5770)	−0.1259 (−0.7234)
*N*	49	44	49	44
*R*-squared	0.5512	0.6362	0.5718	0.6120
Adj *R*-squared	0.4746	0.5655	0.4987	0.5366

***, **, and * indicate significance at 1, 5, and 10% levels, respectively, and those in () are the t-values of the corresponding test statistics. X_3_^*a^ is the government stringency index as of January 27, 2021.

Table 7 illustrates that whether the period from the first case reported to 27 January 2021 is selected as the study period (Models 1 and 2) or 28 January 2021 to 6 August 2021 is selected as the study period (Models 3 and 4), under both no replacement of explanatory variables (Models 1 and 3) and replacement of explanatory variables (Models 2 and 4), religious belief and individual freedom both significantly increase COVID-19 prevalence, while government satisfaction significantly decreases COVID-19 infection rate. From the above findings, it can be concluded that changing the time point of the study do not change the findings of the previous study. The direction of sign of the three value variables (or replacement variables) is fully consistent with the results of the baseline regression and the regression coefficients are all highly significant. This indicates that the regression results are robust.

### Heterogeneity analysis

In order to explore the heterogeneity in the influence of national values on COVID-19 prevention and control across countries and regions with different COVID-19 testing policies, this manuscript introduces the variable COVID-19 testing policy for group regression. According to Our World in Data, there are four categories of COVID-19 testing policies in various countries and regions of the world. They are no testing, testing only for specific populations with COVID-19 symptoms, testing for all people exhibiting COVID-19 symptoms and open public testing (i.e., testing for the whole population). Due to the small amount of data, the testing policy is further divided into two categories in this manuscript. Those offering open public testing are grouped into a high detection rate category and assigned a value of 1. Those offering no testing, testing only for specific populations with COVID-19 symptoms and testing for all people exhibiting COVID-19 symptoms are grouped into a low detection rate category and assigned a value of 0. The regression results are shown in Table 8.

**TABLE 8:** Regression results for grouping of COVID-19 testing policies.

Variable	High detection rate		Low detection rate	
	Model 1	Model 2	Model 3	Model 4
*reli*	1.5925*** (4.4007)		−0.1680 (−0.1561)	
*gove*	−0.7767*** (−3.3905)		−0.6437 (−1.3627)	
*free*	0.8536** (3.1374)		0.0011 (0.0023)	
reli*		0.0633*** (3.6670)		0.0497 (1.3145)
gove*		−0.0236* (−1.9550)		−0.0726* (−1.9866)
free*		1.9723*** (3.0707)		0.5506 (0.5805)
*X*_1_	0.0952*** (3.3383)	0.0906*** (3.3465)	−0.0080 (−0.0909)	−0.0030 (−0.0427)
*X*_2_	1.3157 (0.5801)	1.4869 (0.5942)	7.5725 (1.4600)	10.2216* (2.0150)
*X*_3_	0.0298** (2.4333)	0.0384** (2.6854)	0.0554* (1.7776)	0.0365 (1.2026)
*X*_4_	−0.3058** (−2.2804)	−0.0553 (−0.3739)	−0.2667 (−0.3435)	0.1645 (0.2300)
*N*	29	27	20	17
*R*-squared	0.7782	0.7582	0.4395	0.6494
Adj *R*-squared	0.7043	0.6691	0.3125	0.3768

***, **, and * indicate significance at 1, 5, and 10% levels, respectively, and those in () are the t-values of the corresponding test statistics.

Table 8 shows that whether regressions are selected without replacing explanatory variables (Models 1 and 3) or replacing explanatory variables (Models 2 and 4), the regressions show that the effect of national values on the effectiveness of COVID-19 prevention and control is not significant overall when a country or region has a COVID-19 detection policy with low detection rates or do not have a COVID-19 detection policy. The effect of national values on epidemic prevention and control is generally more significant when a universal COVID-19 testing policy is implemented. Both religious belief and individual freedom significantly increase COVID-19 prevalence, while government satisfaction significantly decreases COVID-19 prevalence under high testing rate policies. This may be due to the fact that in countries and regions with low detection rate policies, governments often do not adopt effective COVID-19 prevention and control policies (Cao et al., 2020). Therefore, the issue of national acceptance and implementation of COVID-19 prevention and control policies does not exist in these countries, resulting in a non-significant effect of national values on COVID-19 infection rates. In countries and regions with high detection rate policies, COVID-19 prevention and control systems are often well established. At this point, the effectiveness of COVID-19 prevention and control depends to a large extent on national recognition and implementation of COVID-19 prevention and control policies. Therefore, national values have a significant impact on the prevalence of COVID-19 infection.

## Conclusion and policy recommendations

Based on the WVS-7 and HFI report in 2020, this manuscript extracts three value variables. They are religious belief, government satisfaction, and individual freedom. This manuscript then constructs an econometric regression model to investigate the impact of these three values variables on the prevention and control of COVID-19 and conducts a heterogeneity analysis.

The study finds that religious belief and individual freedom significantly increase COVID-19 infection rates and are detrimental to COVID-19 prevention and control, while government satisfaction significantly decreases COVID-19 infection rates and contributed to COVID-19 prevention and control. This finding remains robust after introducing instrumental variables, replacing core variables, and changing the time point of the study.

In addition, the impact of national values on the effectiveness of COVID-19 prevention and control varies significantly across countries and regions with different COVID-19 testing policies. Only in countries and regions with high testing rate policies and more developed COVID-19 prevention and control systems do national values significantly influence the effectiveness of COVID-19 prevention and control. Based on the above findings this manuscript makes the following policy recommendations.

First, the government should appropriately strengthen public opinion and emotional guidance on COVID-19 prevention and control policies. This study shows that national values have a significant impact on the effectiveness of COVID-19 prevention and control. In the traditional policy-making process, the government tends to focus more on improving objective economic conditions, while guiding the subjective emotions of the people is relatively absent. Therefore, when the government introduces a series of COVID-19 prevention and control policies, it should strengthen the guidance of publicity and public opinion. The government can disseminate the relevant policies to the public, answer their questions and advocate their active cooperation. We should believe that if citizens truly understand and agree with the country’s prevention and control policies of COVID-19, their recognition and implementation of relevant measures will most likely be greatly improved, and the epidemic prevention and control work will achieve better results.

Second, the government should strengthen, as appropriate to national conditions, the propaganda and guidance of religious people and believers. This manuscript shows that countries and regions with a high level of religious observance will likely result in relatively high rates of COVID-19 infection rate. This indicates that there is some deviation between religious practice and COVID-19 prevention and control in most countries and regions. Therefore, the government should actively provide training to the religious community and the faithful. The government should guide them to raise their awareness of scientific protection, so that the religious community can play a more active role. At the same time, religious places of worship should improve their internal prevention and control measures and resolutely implement the national COVID-19 prevention and control policy. They should establish an organizational structure and develop an emergency mechanism for the prevention and control of the epidemic. They should also clarify the emergency handling process for epidemic prevention and control and do a good job of handling abnormal situations.

Third, the government should strive to improve the nation’s satisfaction with the government and enhance the nation’s sense of access, happiness, and security. The research in this manuscript shows that an increase in national satisfaction with government helps COVID-19 prevention and control to a certain extent. This suggests that controlling COVID-19 requires a relationship of mutual trust between the government and the people, and a mutual agreement on the expectations of the actions to be taken. Therefore, at a time when COVID-19 is rampant, the government should place the utmost importance on protecting the safety of its citizens. To achieve this goal, the government should strengthen the health care system so as to enhance the capacity of health care services. In addition, the government needs to play a role in providing assistance to people’s lives, ensuring that information and data are open and transparent, providing universal access to virus testing and vaccines, preparing personal protective equipment, protecting vulnerable groups, and providing mental health counseling in order to increase people’s satisfaction with the government.

Fourth, the government should correct the deformed view of individual liberalism and promote the correct values of collectivism. This manuscript shows that individual liberalism, with an overwhelming focus on personal interests, has significantly increased the rate of infection and mortality of COVID-19 and exacerbated the spread of the epidemic in some countries and regions. In order to effectively curb the spread of COVID-19, the government should, on the one hand, strengthen education and awareness among the population so that they have a proper understanding of freedom. In addition, if the spread of the epidemic or other serious consequences are caused by excessive individual freedom, serious disciplinary action should be taken against those involved. The government should also take urgent preventive and control measures. On the other hand, the government should also promote the right values of collectivism and enable the nation to firmly establish a sense of the bigger picture. Only in this way can we fight this ideological battle of initiative and protect the COVID-19 prevention and control efforts.

Although this article has extensively explored the relationship between national values on the prevention and control of the COVID-19, it still has certain limitations and deserves subsequent studies. It is noteworthy that this study is purely empirical, but the mechanisms is also important. Therefore, further research could build theoretical models to explain the empirical findings of this research. In addition, if more precise panel data for the countries are available, they can more accurately reflect the relationship between national values on the prevention and control of the COVID-19, and more accurate results and conclusions can be obtained.

## Data availability statement

Publicly available datasets were analyzed in this study. This data can be found here: The seventh wave of World Values Survey (2017–2020) (WVS-7, 2020).

## Author contributions

YL: conceptualization, resources, supervision, project administration, and funding acquisition. YW: methodology, investigation, and data curation. YW and JZ: software. YL and YW: validation. JZ: formal analysis and writing—review and editing. JZ and YL: visualization. YL, JZ, and YW: writing—original draft preparation. All authors contributed to the article and approved the submitted version.

## Footnotes

1 Source: Johns Hopkins University CSSE COVID-19 Data. Due to limited testing, the number of confirmed cases is lower than the true number of infections. For some countries the number of confirmed deaths is much lower than the true number of deaths. This is because of limited testing and challenges in the attribution of the cause of death.

2 On 6 August 2021, Cumulative confirmed COVID-19 cases worldwide exceed 200 million for the first time. This timeline has been chosen for ease of analysis.

3 If the value of the KMO statistic is greater than 0.7, it means that it is middling for factor analysis. Statistics between 0.6 and 0.7 indicate that factor analysis is mediocre. Statistics between 0.5 and 0.6 means that it is miserable for factor analysis, and statistics less than 0.5 means unacceptable (Kaiser and Rice, 1974).

4 This value is calculated from the average value of regression coefficients of geographical location (*free**) of model 2 and model 3 in Table 5, i.e., (1.2837 + 1.4840)/2 = 1.38385.

5 This value is calculated from the average value of regression coefficients of geographical location (*free**) of model 3 and model 4 in Table 6, i.e., (1.7216 + 1.9441)/2 = 1.83285.
